# School attendance matters: co-occurring trajectories of school attendance and academic achievement from elementary to secondary school in a Canadian context

**DOI:** 10.3389/frcha.2026.1729744

**Published:** 2026-03-03

**Authors:** Heather Brittain, Tracy Vaillancourt

**Affiliations:** 1 Brain and Behaviour Laboratory, Counselling Psychology, Faculty of Education, University of Ottawa, Ottawa, ON, Canada; 2 School of Psychology, Faculty of Social Sciences, University of Ottawa, Ottawa, ON, Canada

**Keywords:** academic achievement, adolescence, grade point average (GPA), school absenteeism, school attendance, school transitions, longitudinal study

## Abstract

**Introduction:**

School attendance and academic achievement are central indicators of student engagement and success, and relations between constructs are typically framed as predictors and outcomes, not as co-developing intertwined trajectories across multiple years of schooling. In Canada, where national attendance data are scarce, little is known about how absences and grades co-evolve through critical educational transitions. We examined heterogeneous joint trajectories of school absences and grade point averages (GPAs) from mid-elementary through secondary school (Grade 5 to Grade 12) and examined the transition to secondary school (Grade 8 to Grade 9) as a developmental turning point. We also assessed correlates of academic functioning classes including demographic factors, elementary school type (K-8 vs. middle school), bullying victimization, and depression and anxiety symptoms.

**Methods:**

Data were drawn from 701 students (53% girls; *M*_age_ = 10.9 years) participating in an eight-year longitudinal study spanning Grades 5–12 in southern Ontario, Canada. Official school-record data on annual absences and GPAs were analyzed using parallel-process piecewise latent growth curve modelling and multi-trajectory (latent class) analysis to identify distinct patterns of co-development and discontinuities at the secondary school transition.

**Results:**

On average, absences increased, and GPAs remained relatively stable across Grade 5 to Grade 12, with a significant increase of 2.76% percent days absent at the secondary school transition. Six joint trajectories were identified: three stable groups showed minimal, developmentally appropriate absences with high, moderate, and mid-range GPA and three risk groups showed increasing or chronic absences paired with declining grades (high increasing absences with moderate declining GPA; low-elementary high-secondary absences with low mid-range declining GPA; chronic absences with mid-range declining GPA). Lower socioeconomic status, male gender, and higher depressive symptoms characterized at-risk trajectories.

**Discussion:**

Findings indicate that school attendance and achievement are dynamically linked, and for some, change sharply during the secondary school transition. Results underscore the need for early, targeted monitoring of both domains and for additional research to guide intervention and policy in the Canadian education context.

## 1 Introduction

Positive academic functioning is a cornerstone of prosperous development. School attendance and academic achievement (grades, exams, and standardized test scores) are two aspects of academic functioning that are consistently associated [e.g., (1–5)]. Difficulties in either area signal risk for poorer educational outcomes: students with frequent school absences or low academic achievement are less likely to graduate on time (5, 6), more likely to drop out of high school (7), and less likely to pursue or complete higher education (3, 8, 9). These academic challenges also forecast a range of later adverse outcomes [see (10, 11)], including teen pregnancy (12), adult depression (13), substance use (14–16), incarceration (17), and suicidality (18–20). Given the lifelong implications of problematic academic functioning, understanding how school attendance and academic achievement evolve across development is essential for identifying when and for whom support is most needed.

Although several terms are used to characterize school absences, school attendance typically refers to students’ physical presence in compulsory schooling, and absenteeism includes excusable and inexcusable absences from compulsory schooling (10, 11). Student absences at or above 10% of instructional days is commonly referred to as chronic absenteeism (21, 22). Academic achievement refers to students’ performance on school-based evaluations such as course grades, cumulative grade point averages (GPAs), and standardized test scores (23). These two domains are conceptually distinct yet empirically intertwined indicators of academic functioning.

One context in which academic functioning may be vulnerable is during school transitions, which occur in education systems worldwide (23). In Canada, some students experience a middle school transition in Grade 6 or Grade 7, and most students transition to secondary school between Grade 7 and Grade 10, depending on the province. Compared to elementary school, which offers consistent structure and support, the transition to secondary school can feel unfamiliar and unpredictable. Students must navigate larger environments, shifting schedules, greater academic demands, increased autonomy, and changes in peer groups, often with less adult oversight (24–27). These challenges can heighten students’ concerns and stress as they enter a new phase of schooling (28, 29).

Cross-sectional and short-term longitudinal studies indicate that students often show increased absences and declines in grades or test scores immediately following the transition to secondary school (6, 30–32). However, little is known about how these short-term disruptions fit within students’ long-term developmental trajectories. Although many countries have prioritized monitoring student attendance [e.g. (33, 34),], Canadian data are limited. As outlined by Whitley et al. (35), there are several contributing factors, including (1) lack of federal oversight; (2) limitations of national cross-sectional research studies that include only single age groups (e.g., PISA; Grade 8 students (36); (3) prior national longitudinal studies included single items on days skipped [e.g., NLSCY (37)]; and days attended of the last 7 days [e.g., CHSCY, 2023 (38)], with ongoing surveys [e.g., CHSCY, 2025 (39)] omitting school attendance; and (4) until recently, absenteeism has not been a research focus of Canadian education scholars (40).

In the current study, we jointly modeled school absences and academic achievement in a large Canadian cohort followed annually from Grades 5 through 12. This design allowed us to examine how attendance and grades co-developed across elementary and secondary school, whether the transition to secondary school marked a discontinuity in these trajectories, and which individual and contextual factors differentiated adaptive vs. maladaptive pathways.

### 1.1 School absences over time and across school transitions

School absences are typically characterized by a ‘U’ pattern, with high absences in pre-school/early elementary years, generally low and stable rates in elementary grades, and rise sharply in secondary school, largely driven by increasing unexcused absences (1), a pattern documented in cross-sectional reports from national governments (41), school boards (42), and research organizations (43). Longitudinal analyses replicate these trends, showing either stable (30) or slight declines in attendance and increases in unexcused absences across mid-elementary grades (44, 45), and steeper increases during secondary school (1, 30, 45).

Although these reports provide population averages, researchers have used person-centered analytic approaches, like latent class growth analysis and growth mixture modeling, to study heterogeneity in school absenteeism longitudinally over time to classify students into subgroups based on similar patterns of attendance. This approach has been done with data from American, Finnish, and British students examining different periods of compulsory schooling including early elementary school (46, 47), middle-school (48), across full elementary [K-8; (49)], middle through secondary school [Grades 6-12; (50)], and one study across full compulsory education [England, Years 1-11; (1)].

Across studies, researchers have consistently identified between three and six trajectory classes of school absences, with notable developmental shifts from elementary into secondary school. In the elementary and middle school years (46–50), most students (46%–90%) follow a low-stable absence trajectory (i.e., few absences), while smaller groups (5%–30%) show moderate-stable, decreasing, or gradually increasing absences, and a minority (1%–8%) demonstrate chronic high or early-onset high absences. By secondary school, however, heterogeneity becomes more pronounced. Benner and Wang (50)^1^ reported that approximately one-third of students maintained low absences, while the remainder showed moderate-to-high and increasing absences, and Dräger et al. (1) identified five distinct classes spanning compulsory education, with two large groups showing consistently low (66%) or moderate authorised absences (28%), and smaller groups following increasing unauthorised or authorised absences (6% combined). These findings suggest that although most students experience consistently low levels of school absenteeism, risk for moderate and chronic absences grows during the upper schooling years, particularly in subgroups whose absences escalate over time.

These developmental shifts are evident, yet few studies have modeled the transition point itself. Benner and Graham (30) demonstrated marked increases in absences across the first two years of secondary school compared to the average slope across two years of middle school; however, they focused on demographic and school structural aspects and did not look at unaccounted-for heterogeneity in patterns. Dräger et al. (1) charted trajectories of absences across elementary and secondary school but did not statistically account for the impact of transition. Benner and Wang (50) did address the transition in overall curves through a piecewise model, allowing different slopes in elementary and secondary school; however, the approach was not carried through in the class analysis where separate middle- and secondary-school models were estimated and the odds of moving class to class were assessed. These approaches prevent a unified test of continuity and create difficulties in testing whether slope changes at Grade 8 to Grade 9 are statistically significant for subgroups of students, and as a result, subtle patterns that unfold across the secondary school transition may be overlooked. Beyond documenting trajectories, absence classes are also differentially associated with academic outcomes.

Students with moderate or increasing school absences show poorer performance across multiple indicators, including standardized test scores in elementary and middle school (46, 47), GPA and graduation in secondary school (50), higher dropout risk (49), and lower performance in General Certificate of Secondary Education, particularly for increasing unauthorized absences (1). In predicting school absences classes, most studies have focused on school-level correlates (e.g., teacher experience, school size) and demographics (e.g., gender, SES) and have not considered individual-level processes, such as bullying victimization or mental health problems, that may shape trajectories. Moreover, even though Benner and Wang (50) adjusted for Grade 8 GPA, they did not model the time-varying interplay between absences and GPA. Considering that GPA and absences are more strongly correlated when measured proximally (5, 8), greater variability in post-transition grades may have been overlooked, and accounting for this interdependence could have altered study conclusions. These limitations highlight the need for models that span elementary through secondary school and explicitly capture how school absences and academic performance co-develop across transitions.

### 1.2 Grades, test scores, and GPA over time and across school transitions

Like absences, researchers have examined average trajectories of academic achievement across compulsory schooling. In elementary years, students display steady gains in reading, math, and core skills (51–54), with middle school students experiencing a temporary disruption (55). The secondary school transition has been shown to be an inflection point; GPAs are stable through middle school but drop sharply at the secondary school transition, after which they level off or decline (30, 32, 53). However, there is variation in students’ academic achievement over time.

Across studies, researchers have typically identified three to four latent classes of academic achievement, with patterns differing across schooling periods. In the elementary years, while most students (50%–75%) follow stable or improving trajectories, patterns among lower-performing students vary by study (52, 54). Fu et al. (52) identified a class with declining achievement (26%) and another class that remained persistently low-achieving (24%), whereas Lu (54) found that the lowest-performing math skills group was characterized by slower, but still positive, growth over time. In a two-year study of Chinese middle (Grade 7) and secondary school (Grade 10) students (56), observed four classes ranging from high-positive (20%), mid-negative (38%), and two low-stable groups (33%, 9%). In a study of upper-year elementary Canadian students, Duchesne et al. (57) identified three academic functioning classes: stable-high (63%), stable-low (23%), and declining (14%). At the start of secondary school, three and four distinct trajectory classes are consistently identified. In the U.S., Bowers and Sprott (58) reported four classes across the first three semesters of secondary school, with the largest groups comprising roughly 19% (high-achieving stable) and 57% each (mid-achieving stable) and two smaller groups (mid-decreasing 11%; low-increasing 14%). In China, Liu and Lu (45) found three classes during the first year of senior secondary school, with a high declining group making up nearly 80% of the sample and the remaining students divided across increasing or normative (moderate-stable) groups. Over a long span across secondary school, researchers have identified three profiles including high (stable or slightly-increasing), moderate declining grades, and low-increasing classes (59, 60).

The inconsistency in trajectory study findings may be due to differences in achievement assessments which are confounded by nation. Most studies of Chinese youth rely on outcomes of standardized exams in Chinese, English, and math (45, 52, 56), whereas studies of American students rely on GPA (58, 59) or single-subject scores [e.g., math; (54)], or subjects analyzed separately in Taiwan (60), and the Canadian study included a 2-item parent-reported composite of academic motivation and achievement (57). Moreover, studies did not consistently span the same academic period and were isolated to elementary (52, 54) or secondary school (58, 59). When middle school and secondary school samples were included in a single study, they were analyzed as one sample (45), precluding an analysis of differences in trajectories of middle and secondary school students. Many studies also span only 1.5–2 years (45, 58), which means we know little about long term patterns. These findings show that while person-centered analyses highlight clear heterogeneity in academic achievement, especially in secondary school, these studies do not explicitly assess how different groups of students change academically across school transitions.

School transitions frequently represent inflection points in academic achievement. Temporary interruptions or declines in grades have been observed following entry into both middle school (55) and secondary school (26, 30). Reyes et al. (31) documented that although students had similar grades pre-transition, the amount of decline immediately following the transition (i.e., minimal, moderate, or major) was associated with their grades and absences across the remainder of secondary school, but the low sample size precluded statistical tests of these hypotheses. In a statewide longitudinal study, Schwerdt and West (61) found that students transitioning to middle school in Grade 6 or 7 experienced sustained achievement declines through Grade 10, whereas those remaining in K–8 schools were more stable. Students entering secondary school in Grade 9 also showed a GPA drop, followed by partial recovery. Vasquez-Salgado and Chavira (32) reported similar findings in Latino youth, noting steep GPA declines across the Grade 8–9 transition, particularly among higher-achieving students, with gender differences in post-transition slopes (girls declining, boys rising). Although informative, most studies rely on averages (assuming a single path for all students) or short-term comparisons (with limited assessments on either side of the transition). Few have used trajectory models with statistical discontinuities across a long span accounting for heterogeneity, leaving unclear whether observed declines reflect developmental changes or transition-linked turning points, and whether subsets of students respond differently.

### 1.3 Grades and absences

Dräger et al. (62) recently found that school absences in the year before secondary school transition and in the 4 years after were most strongly associated with final secondary exam scores and highlighted the importance of the secondary school transition period. Although Benner and Graham (30) examined the impact of school transition on academic functioning using attendance and GPA, these highly correlated areas were looked at separately, and a joint trajectory was not examined, leaving readers to infer associations between the rate of change in increasing absences and declining GPA in secondary school. Grades and school absences are studied separately, but these domains are strongly interconnected. Indeed, national cohort studies consistently show that students who miss more school earn lower grades and test scores and are less likely to pursue postsecondary education (63, 64). This association holds across developmental stages and national contexts, suggesting that achievement and engagement are mutually reinforcing. As discussed above, both absences and grades display various pathways, and students following problematic trajectories often have lower achievement and higher dropout risk (1, 47, 49, 50).

Despite these parallels, to our knowledge, no published study has modeled grades and absences jointly to capture how they co-develop or whether transitions alter their association. Instead, absences are usually treated as predictors or outcomes of achievement [e.g., (1, 5, 62)]. This segmented approach does not address whether subgroups of students experience coupled trajectories. For example, it is unclear if chronic absenteeism predominantly occurs among those on declining GPA pathways, or whether stable attendance buffers grade declines at school transitions. In the current study, we addressed this knowledge gap by applying multi-trajectory modeling to identify co-occurring patterns of grades and absences across Grades 5–12 and tested whether the secondary school transition (Grade 8–9) marked a discontinuity in these joint pathways.

### 1.4 Correlates

A range of individual and contextual factors shape students’ academic trajectories, especially during school transitions. The earlier move to middle school can either foster resilience or act as a sensitizing event that heightens vulnerability to later adjustment difficulties (65, 66). Gender may moderate how students respond to school transition. For example, boys often struggle more with adapting to new expectations than girls (29, 67). On the other hand, Vasquez-Salgado and Chavira (32) found that although both genders showed declines in GPA during the secondary transition, girls’ grades tended to continue declining while boys’ GPA tended to increase across the first two years of secondary school. Socioeconomic status also matters, with achievement gaps between low- and high-SES youth widening after entry into secondary school (68). Transitions coincide with changes in peer relations, including bullying involvement (69), and shifts in internalizing problems such as depression and anxiety (26, 70, 71); these areas of psychosocial functioning are also associated with school absenteeism and poorer achievement (7, 72–75).

### 1.5 Current study

In the present study, we build on prior research by jointly modeling school absences and grades across adolescence (i.e., Grades 5–12) in a large, longitudinal cohort of Canadian students. Although most prior studies have examined these domains separately, our unified approach identified heterogeneous multi-trajectory groups that reflect how absences and grades fluctuate together across adolescence. We also examined whether these joint pathways showed discontinuities at the secondary school transition, addressing a critical gap in the literature where transitions have rarely been modeled as potential turning points. Finally, we examined whether joint trajectory group membership was associated with individual and contextual correlates, including gender, socioeconomic background, bullying victimization, depression and anxiety symptoms, and elementary school structure (i.e., K-8 vs. middle school).

Existing academic studies typically describe pre- and post-transition patterns or identify trajectories across schooling but do not statistically model the transition itself as a discontinuity, leaving it unclear whether observed changes reflect general developmental trends or true transition-linked turning points [see (69), for an example in bullying]. Although multi-trajectory modeling has been increasingly applied in areas such as mental health (71), it has rarely been used to study academic functioning, and no prior work has examined absences and grades in a single model across school transitions. Accordingly, we identified joint trajectories of grades and absences from Grades 5 through 12. Specifically, we tested whether these trajectories demonstrated discontinuities at the secondary school transition and examined if individual and contextual factors differentiated membership in distinct joint trajectory groups.

We predicted that we would identify four to six distinct joint trajectories reflecting different combinations of absences and GPA. Specifically, we anticipated low-stable, moderate-increasing, and high-chronic patterns of absences, with faster increases during secondary school, combined with at least three GPA trajectories (high-stable, moderate-declining, and low-stable). We further predicted overlapping trajectories, such that students with consistently low absences would correspond to high-stable GPA trajectories, whereas those with chronic or escalating absences would cluster in low or declining GPA groups. To support validity, classes were compared on parent-reported absences and standardized achievement test scores with the expectation that at-risk classes would display higher parent-reported days absent and lower standardized achievement scores.

Consistent with prior work, we also predicted that gender and socioeconomic background would differentiate trajectory group membership. Beyond demographic factors, we predicted that poorer psychosocial functioning (i.e., higher bullying victimization and higher depression and anxiety symptoms), examined as averages within elementary and secondary school for parsimony, would be found in academic functioning classes characterized by chronic or increasing absences and low and declining achievement. We also predicted that students experiencing middle school would have a higher likelihood of belonging to at risk groups compared to those in full elementary (K-8) schools (61).

## 2 Materials and methods

### 2.1 Participants

Data were drawn from the McMaster Teen Study, a comprehensive multi-informant, multi-method, longitudinal study of students with a focus on interpersonal relationships, mental health, and academic achievement. In Time 1 of the study (spring 2008), Grade 5 students were recruited from 51 randomly selected schools from one large southern Ontario Public School Board. A total of 1023 students took part in the first year and 875 families agreed to take part in the longitudinal study; 703 participants took part in at least one follow-up between Time 2 and Time 8 (Grade 12), with 701 having data on grades and absences. In the first year of the study, students were on average 10.91 years old (*SD* = 0.36), half were girls (53%), and most were White (76%; 16% racially/ethnically-diverse; 8% missing). Most parents had completed postsecondary education (69.6%), and the reported median household income fell between $70,000–$80,000 CAD, comparable to both the municipal and provincial medians at the time of recruitment (www.statcan.gc.ca).

### 2.2 Procedure

University research ethics and parental consent and student assent were obtained annually. In the first year of the study, survey data were collected from students in schools and in subsequent years at participants’ homes (paper/pencil or online) and parents/guardians completed a telephone interview. Official school records were obtained from the participating school board (Grades 5–12). See Vaillancourt et al. (69) for details.

### 2.3 Measures

#### 2.3.1 Academic functioning

Teacher-reported grades and school absences from Grades 5–12 were obtained from the participating school boards.

##### 2.3.1.1 Days absent

In elementary school, the total number of days absent from school were obtained from school records. In secondary school, the number of classes missed per class were provided from school records; days absent per term was computed by an average of class absences and term absences were summed for a total score. Days absent per year were converted to a percentage score of the total instructional days absent, which were correlated across time, *r*s = .22–.83.

##### 2.3.1.2 Grades

In elementary school, letter grades were obtained for each term (T1–T3, 3 terms; T4, 2 terms^2^) for courses in English, French, math, science (T1–T4), social studies (T1 and T2), and history (T3 and T4). When participants had more than one grade within a subject (e.g., English reading, writing, and oral), grades were averaged prior to computing term averages. In secondary school, percentage scores were obtained for each course and converted to letter grades for consistency with elementary school data. Secondary school courses were diverse in the type of classes students could take and the academic stream. All available course grades were averaged. Grades ranged from <D- = 0 to A + = 12 (D- = 50%–52%; D = 53%–56%; D + = 57%–59%; C- = 60%–62%; C = 63%–66%; C + = 67%–69%; B- = 70%–72%; B = 73%–75%; B + = 76%–79%; A- = 80%–84%; A = 85%–89%; A + = 90%–100%). Correlations between term GPAs within a given year (Grades 5–8) were high, *r*s = .86–.93, as were GPA across years, *r*s = .65–.88. Because of the varied courses that students could take in secondary school, a traditional reliability coefficient could not be computed. Secondary school GPAs from Grades 9–12 were highly correlated, *r*s = .63–.89.

#### 2.3.2 Latent class validation items

##### 2.3.2.1 Parent-reported days absent

In Grades 6 to 12, parents reported the approximate number of days during the school year that their child was absent from school for any reason using a 6-point scale (0 = zero; 1 = 1–3 days; 2 = 4–6 days; 3 = 7–10 days; 4 = 11–20 days; 5 = more than 20 days).

##### 2.3.2.2 Standardized test scores

In Grade 6, students completed Ontario's Education Quality and Accountability Office (EQAO) assessments in reading, writing, and mathematics and in Grade 9 completed a standardized math assessment. These province-wide standardized tests evaluate student achievement against Ontario curriculum expectations (76). EQAO results were obtained from the participating school board. The possible values for EQAO scores were 0.1 (below provincial expectations) to 4.9 (above provincial expectations).

#### 2.3.3 Latent class correlates

##### 2.3.3.1 Demographics

In the first year of the study, student participants reported their gender (coded as: girl = 1; boy = 0). Participants and their parents reported their racial/ethnic background in Time 1 and Time 2 (i.e., White, Middle Eastern-Canadian, African/West-Indian-Canadian, South-Asian-Canadian, Asian-Canadian, Native-Canadian, South/Latin-American-Canadian, and Other). Although the racial/ethnic diversity is representative of the population from which participants were sampled, due to the low frequencies of racially/ethnically diverse groups, race/ethnic background was re-coded as White (coded 0) and racially/ethnically-diverse (coded 1). Household income and parental education were reported by parents in the parent interview in Time 1 (and Time 2 if not reported in Year 1). Income was reported using 8 categories (1 < $20,000, 2 = 20,000–30,000, 3 = 30,000–40,000, 4 = 40,000–50,000, 5 = 50,000–60,000, 6 = 60,000–70,000, 7 = 70,000–80,000, 8 > 80,000) and the highest level of education attained was reported using 5 categories (1 = Did not complete secondary school, 2 = Completed secondary school, 3 = College diploma or trades certificate, 4 = University undergraduate degree, 5 = University graduate degree).

##### 2.3.3.2 Elementary school type

In elementary school, some students were enrolled in a kindergarten to Grade 8 school (coded 0), and others experienced a middle school transition in Grade 6 or Grade 7 (coded 1).

##### 2.3.3.3 Mental health symptoms

Depression and anxiety symptoms were measured from Grade 5 to Grade 12 using the Behavior Assessment System for Children-2 [BASC-2 (77)], with the child version administered in Grades 5 and 6 and the adolescent version in Grades 7–12. Items were rated on a 4-point scale (0 = *never* to 3 = *almost always*) or dichotomously (0 = *false*, 2 = *true*). Items that were common to both versions were used in the present study, following procedures by Krygsman and Vaillancourt (78). Subscales included symptoms of depression (12 items) and anxiety (10 items). Scales demonstrated excellent internal consistency (depression *α* = .87–.91; anxiety *α* = .85–.91). Items within subscales were summed within year and then averaged over schooling period to create composite scores for elementary and secondary school.

##### 2.3.3.4 Bullying victimization

In Grade 5 to Grade 12 participants completed the Vaillancourt and Hymel Bullying Involvement Scale that assessed bullying victimization and perpetration (79, 80). Participants read a definition of bullying before reporting how often in the last 3 months that they have experienced being bullied. Five items were asked including a general question, physical, verbal, social, and cyber bullying, with several examples of what the targeted behaviour entailed. Youth responded using a 5-point frequency scale (0 = *never*, 1 = *only once or twice*, 2 = *2 or 3 times a month*, 3 = *once a week*, 4 = *several times a week*). Items were averaged to create annual composite scores and had good internal consistency (*α* = .79–.82). Elementary school (Grade 5 to Grade 8) and secondary school (Grade 9 to Grade 12) scores were averaged, respectively.

### 2.4 Statistical analysis

#### 2.4.1 Descriptive statistics and missingness

Participants in the analytic sample were compared to non-participants based on Grade 5 demographic variables (i.e., gender, ethnicity, household income, parent education) and Grade 5 days absent and GPA. Group comparisons were conducted using two-tailed independent-samples *t*-tests and chi-square tests of independence. Little's MCAR test was applied to assess the randomness of missing data in the analytic sample. Mplus was used to derive estimated means and correlations for absences and GPA using full information maximum likelihood estimation with robust standard errors to account for missing data and non-normality.

#### 2.4.2 Average growth curves

Mplus version 8.11 (Muthén and Muthén, 2017) was used to estimate parallel-process piecewise latent growth curve models for GPA and absences, applying full information maximum likelihood to address missing data, with robust standard errors (i.e., maximum likelihood robust estimation; MLR) to account for deviations from normality. Consistent with Rioux et al. (113), the models incorporated level and slope discontinuities, enabling trajectories to shift around a specified event. The transition to secondary school was modeled as this event. Growth parameters for grades and absences were correlated. Each outcome included two segments: Grades 5–8 (pre-transition) and Grades 9–12 (post-transition), both specified with linear and quadratic parameters to capture potential nonlinear change. A level discontinuity at Grade 9 allowed for a level change in trajectories between Grades 8 and 9. Models included covariance terms between latent curve factors. Model adequacy was assessed using conventional fit statistics, with criteria of CFI ≥ 0.95, RMSEA ≤ 0.06, SRMR ≤ 0.08, and a nonsignificant *χ*^2^ test indicating acceptable fit (81).

We further examined if elementary and secondary school curves had a similar shape by setting slope and quadratic parameter estimates equal and testing equality using the Wald chi-square test. We also examined if there was a marked change in absences and grades immediately before and after the secondary school transition by computing estimated scores in Grade 8 and Grade 9 and testing for equality using the Wald chi-square test.

#### 2.4.3 Heterogeneous classes

Parallel-process Latent Class Growth Analysis [LCGA; also referred to as Group-Based Multi Trajectory Modeling, GBMTM (82)] was applied to identify unobserved subgroups of students who shared similar developmental patterns in absences and grades. Model fit was evaluated using the Bayesian Information Criterion (BIC), the Lo–Mendell–Rubin Likelihood Ratio Test (LMR-LRT), and the Bootstrapped Likelihood Ratio Test (BLRT), with classification quality supported by posterior probabilities greater than 0.70. Final models were selected based on statistical fit, theoretical clarity, and adequate group size (≥2% of the sample) with trajectory shapes consistent with the existing literature. Models with up to nine classes were estimated. Trajectory estimates at Grade 8 and Grade 9 were compared to evaluate changes around the transition to secondary school.

#### 2.4.4 Class validation

To substantiate class configuration, we examined differences in parent-reported absences across time from Grade 6 to Grade 12 and standardized test scores in Grade 6 (i.e., reading, writing, math) and Grade 9 (i.e., math) using ANOVA and *post hoc* pairwise comparisons.

#### 2.4.5 Class correlates

After identifying trajectory classes, we examined whether they were differentially associated with hypothesized correlates, including gender, socioeconomic background, and mental health functioning in elementary and secondary school. Preliminary chi-square tests and ANOVAs were conducted, and only correlates that were statistically significant at *p* < .05 were probed, using Fisher's Least Significant Difference test where homogeneity of variance tests were not rejected, and Dunnett's T3 where the test of homogeneity of variance was rejected. The Benjamini–Hochberg correction was applied to omnibus and *post hoc* tests to control for multiple comparisons (83).

#### 2.4.6 Sensitivity analysis

A sensitivity analysis was conducted to examine whether the class structure could be replicated using a subset of participants that had data in both elementary school and in secondary school (*n* = 573).

## 3 Results

### 3.1 Participants

Of the 875 participants enrolled, 701 had data on absences/grades and comprised the analytic sample. Compared to participants not retained for longitudinal analyses, the analytic sample had higher parental education [*t*(805) = −6.261, *p* < .001], household income [*t*(770) = –6.060, *p* < .001] and were more likely to be White [82.4% vs. 64.3%; *χ*^2^ (1, *N* = 787) = 22.785, *p* < .001]. No significant differences emerged between included and excluded participants on sex [*χ*^2^ (1, *N* = 875) = 0.037, *p* = .848]. Those in the analytic sample differed on Grade 5 absences [*t*(801) = 3.997, *p* < .001] and GPA [*t*(829) = −6.303, *p* < .001]. Little's MCAR test suggested that data in the analytic sample were not missing completely at random [*χ*^2^(880) = 1658.006, *p* < .001]. Missingness on absences and grades in later waves was associated with earlier observed higher absences and lower grades. Consistent with standard practice (84), data were treated as missing at random using full information maximum likelihood (FIML) estimation in analyses.

### 3.2 Descriptive statistics and correlations

Table 1 includes the descriptive statistics for school absences and grades across time, as well as for correlates. Within-time correlations between absences and grades (*r*s = −.619 to −.126, *p*s < .001) and across-time associations were statistically significant, ranging from low to moderate (*r*s = −.573 to −.102, *p*s < .05). Stability across adjacent time points was high for absences (*r*s = .542 to .831, *p*s < .001) and grades (*r*s = .773 to .887, *p*s < .001).

**Table 1:** Descriptive statistics and correlations among annual scores for percent days absent from school and GPA in grades 5 through 12.

	*M*	*SD*	Correlations															
Variable			1	2	3	4	5	6	7	8	9	10	11	12	13	14	15	16
Percent days absent																		
1. Grade 5	3.77	3.57	1															
2. Grade 6	3.99	3.57	.589	1														
3. Grade 7	5.03	4.46	.461	.542	1													
4. Grade 8	5.57	5.13	.436	.509	.641	1												
5. Grade 9	8.51	8.48	.281	.375	.455	.564	1											
6. Grade 10	7.83	7.85	.270	.332	.403	.484	.831	1										
7. Grade 11	13.59	11.75	.243	.306	.303	.410	.625	.690	1									
8. Grade 12	15.96	13.39	.221	.254	.247	.382	.593	.651	.777	1								
Grade point average																		
9. Grade 5	8.11	1.58	*−.126*	*−*.152	*−*.133	*−*.145	*−*.259	*−*.297	*−*.336	*−*.294	1							
10. Grade 6	8.29	1.49	*−.112*	*−*.185	*−*.124	*−*.134	*−*.240	*−*.274	*−*.297	*−*.286	.827	1						
11. Grade 7	8.16	1.93	*−.119*	*−*.213	*−*.168	*−*.181	*−*.304	*−*.327	*−*.339	*−*.318	.767	.827	1					
12. Grade 8	8.49	2.16	*−.102*	*−*.200	*−*.222	*−*.261	*−*.350	*−*.378	*−*.378	*−*.378	.726	.792	.875	1				
13. Grade 9	8.55	2.55	*−.118*	*−*.184	*−*.242	*−*.255	*−*.560	*−*.534	*−*.516	*−*.501	.654	.661	.721	.773	1			
14. Grade 10	8.25	2.95	*−.136*	*−*.204	*−*.253	*−*.249	*−*.573	*−*.619	*−*.562	*−*.545	.652	.642	.713	.755	.887	1		
15. Grade 11	7.83	3.04	*−.117*	*−*.185	*−*.181	*−*.212	*−*.469	*−*.504	*−*.596	*−*.579	.629	.622	.664	.692	.821	.835	1	
16. Grade 12	8.14	3.02	*−.108*	*−.142*	*−*.171	*−*.180	*−*.394	*−*.420	*−*.505	*−*.583	.553	.559	.586	.622	.735	.763	.836	1

Estimated means, standard deviations, and correlations from Mplus (using full information maximum likelihood estimation). Correlations in italics significant at *p* < .05. All other correlations significant at *p* ≤ .001.

### 3.3 Average trajectories—parallel-process piecewise latent growth curve models

#### 3.3.1 Model fit

**Figure 1:**
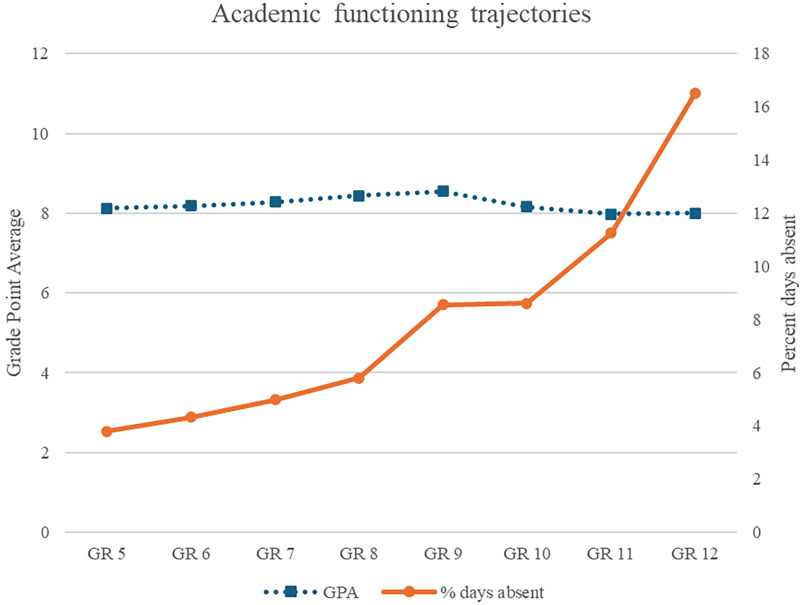
GR, grade; GPA, grade point average. Percent days absent and GPA parallel-process piecewise latent growth curve model with level and curve discontinuity. Grades 5–8 are elementary school and Grades 9–12 are secondary school.

Parameter estimates for the discontinuity level and curve parallel-process latent growth models for grades and absences are presented in Table 2. The preliminary model yielded a non-positive definite residual covariance matrix. To remedy this, the quadratic variance components were successively constrained to 0 until the model ran without error (variance of elementary and secondary school quadratic parameters for absences and GPA constrained to 0). The resulting model fit was acceptable, *χ*^2^(88) = 312.010, *p* < .001, CFI = .950, RMSEA = .060 (90% CI = .053–.068), SRMR = .075 (see Figure 1). The intercept, slope, and level parameters had statistically significant variance estimates. In this model, the intercepts of percent days absent and GPA were negatively correlated, *r* = -.203, *p* *<* .001, as were slope factors in elementary, *r* = -.465, *p* *<* .001, and secondary school, *r* = -.637, *p* *<* .001. The secondary school level change factors for absences and GPA were also correlated, *r* = -.661, *p* *<* .001, with larger increases in absences associated with larger decreases in GPA.

**Table 2:** Parameter estimates for absences and GPA parallel-process discontinuity latent growth curves.

Parameter	Estimate	s.e.	*p*
% days absent			
Intercept	**3.800**	**0.139**	**<.001**
Slope 1	**0.452**	**0.181**	**.013**
Quadratic 1	0.071	0.060	.233
Slope 2^a^	**−1.261**	**0.340**	**<.001**
Quadratic 2	**1.303**	**0.133**	**<.001**
Event—secondary school	**4.763**	**0.375**	**<.001**
GPA			
Intercept	**8.128**	**0.060**	**<.001**
Slope 1	0.030	0.042	.474
Quadratic 1	0.024	0.014	.079
Slope 2	**−0.499**	**0.074**	**<.001**
Quadratic 2	**0.106**	**0.026**	**<.001**
Event—secondary school	**0.419**	**0.084**	**<.001**

GPA, grade point average. Level and slope discontinuity latent growth curve models. Slope 1 and quadratic 1 are parameter estimates representing the average elementary school (Grades 5–8) curve and slope 2 and quadratic 2 are parameter estimates representing the average secondary school (Grades 9–12) curve. Event is for the additional level change associated with secondary school. Covariance terms between latent curve factors also estimated in the models. Statistically significant parameter estimates are bolded.

^a^ The negative slope reflects the initial rate of change at Grade 9, whereas the quadratic term dominates the overall trajectory shape; linear and quadratic parameters should be interpreted jointly.

#### 3.3.2 Secondary school transition

The elementary and secondary school portions of the absences trajectory were not equal, *χ*^2^(2) = 101.329, *p* < .001, and there was an increase of 2.76% of instructional days between Grade 8 and Grade 9, *χ*^2^(1) = 65.918, *p* < .001. In elementary school, the average absences curve was slightly increasing, with a statistically significant increase across the secondary school transition, and the secondary school average curve increased at a higher rate than in elementary school.

The elementary and secondary school portions of the GPA trajectory were not equal, *χ*^2^(2) = 68.687, *p* < .001. In elementary school, the average GPA curve was flat, whereas in secondary school, the average GPA curve was decreasing with a slight rebound in Grade 12. The Grade 8 and Grade 9 average estimated GPA scores were not different, *χ*^2^(1) = 2.262, *p* = .133.

### 3.4 Heterogeneous classes: parallel-process latent class growth models

**Figure 2:**
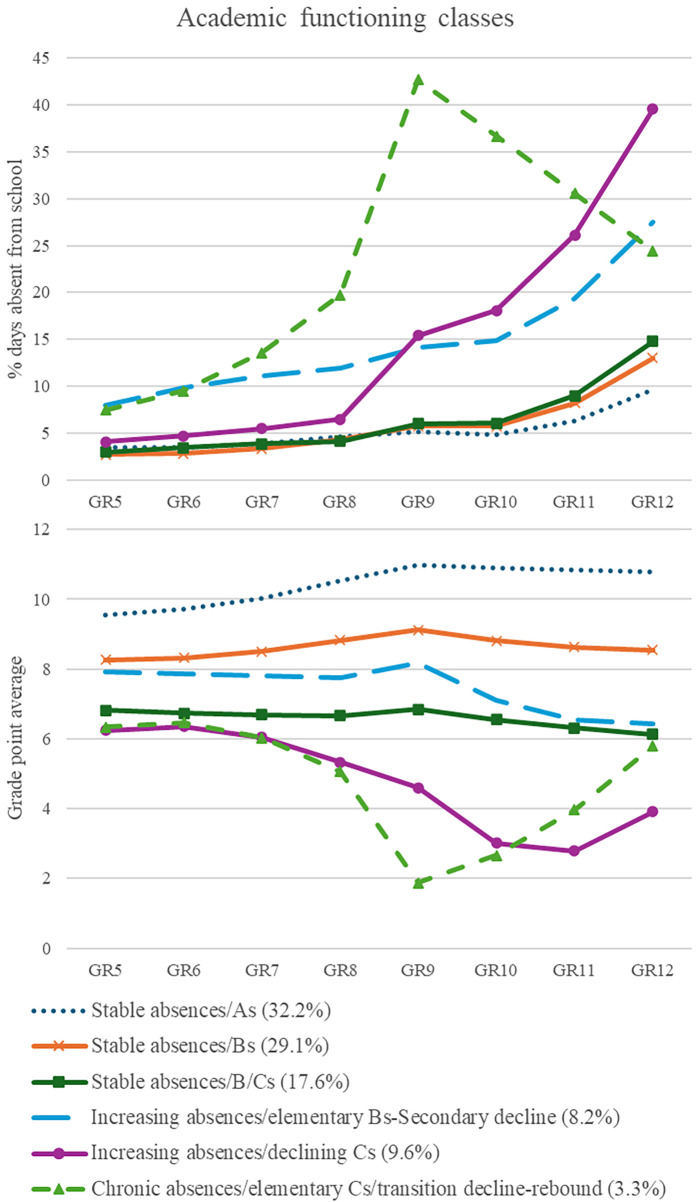
GR, grade; GPA, grade point average. Percent days absent and GPA parallel-process latent class growth analysis six class solution. Grades 5–8 are elementary school and Grades 9–12 are secondary school.

The seven, eight, and nine-class models did not replicate the best log-likelihood, even with increased random starts. These higher-order models consistently identified a class of three individuals. To aid in model identification, these cases were then excluded from analysis and all solutions were re-run. Fit indices are shown in Table 3. The lowest log-likelihood was not replicated for the nine-class solution and was not considered a viable solution. BIC declined steadily from the two- through eight-class solutions. All models produced posterior probabilities above .70, entropy between .884 and .901, and significant BLRT values. The seven- and eight-class solutions did not add conceptual clarity beyond the six-class solution. The six-class model had adequate group sizes and yielded conceptually meaningful distinctions. Starting values from this model were used to test a model with twice the random starts to ensure the lowest loglikelihood value was replicated. For reasons of model fit, parsimony, and interpretability, the six-class solution was selected as the final latent class growth model (see Figure 2; Table 4).

**Table 3:** Fit indices for latent class for absences and GPA.

No. of Groups	BIC	LMR-LRT	BLRT	Entropy
Co-occurring depression and anxiety symptoms				
1 Class	48,192.902		–	–
2 Class	45,256.548	<.001	<.001	0.901
3 Class	44,211.088	.014	<.001	0.895
4 Class	43,713.629	.256	<.001	0.897
5 Class	43,248.892	.437	<.001	0.906
6 Class	**42,948.251**	**.121**	**<.001**	**0.886**
7 Class	42,741.257	.569	<.001	0.884
8 Class^a^	42,533.737	.746	<.001	0.889
9 Class^a^	Best loglikelihood not replicated			

BIC, bayesian information criterion; LMR-LRT, Lo-Mendell-Rubin likelihood ratio test; BLRT, bootstrapped likelihood ratio test. Bold are the final selected class solutions.

^a^ Best log likelihood not replicated.

**Table 4:** Parameter estimates for percentage days absences and GPA parallel-process discontinuity latent class growth curve analysis.

		% days absent			GPA		
Parameter	*n/%*	*Estimate*	*s.e.*	*p*	*Estimate*	*s.e.*	*p*
Class 1 stable absences/As							
	225/32.2%						
Intercept		**3.482**	**0.239**	**<.001**	**9.558**	**0.078**	**<.001**
Slope 1		−0.161	0.260	.536	0.076	0.060	.203
Quadratic 1		**0.175**	**0.080**	**.028**	**0.082**	**0.018**	**<.001**
Slope 2		**−1.224**	**0.327**	**<.001**	−0.085	0.062	.168
Quadratic 2		**0.913**	**0.106**	**<.001**	0.005	0.021	.814
Event—secondary school		**1.612**	**0.303**	**<.001**	**1.424**	**0.074**	**<.001**
Class 2 Stable absences/Bs							
	203/29.1%						
Intercept		**2.690**	**0.228**	**<.001**	**8.267**	**0.119**	**<.001**
Slope 1		−0.081	0.239	.734	−0.016	0.080	.837
Quadratic 1		**0.201**	**0.085**	**.019**	**0.068**	**0.024**	**.005**
Slope 2		**−1.196**	**0.468**	**.011**	**−0.364**	**0.149**	**.015**
Quadratic 2		**1.205**	**0.176**	**<.001**	0.057	0.051	.258
Event—secondary school		**3.057**	**0.420**	**<.001**	**0.856**	**0.147**	**<.001**
Class 3 stable absences/B/Cs							
	123/17.6%						
Intercept		**2.970**	**0.285**	**<.001**	**6.812**	**0.168**	**<.001**
Slope 1		0.546	0.303	.072	−0.083	0.142	.558
Quadratic 1		−0.057	0.104	.582	0.012	0.040	.771
Slope 2		−1.330	0.984	.177	−0.323	0.219	.141
Quadratic 2		**1.418**	**0.508**	**.005**	0.028	0.085	.738
Event—secondary school		**3.015**	**0.664**	**<.001**	0.032	0.244	.895
Class 4 increasing absences/elementary Bs-high school decline							
	57/8.2%						
Intercept		**8.009**	**1.129**	**<.001**	**7.934**	**0.245**	**<.001**
Slope 1		**2.053**	**0.978**	**.036**	−0.075	0.133	.574
Quadratic 1		−0.249	0.323	.441	0.005	0.04	.908
Slope 2		−1.052	1.623	.517	**−1.298**	**0.407**	**.001**
Quadratic 2		**1.842**	**0.738**	**.013**	0.239	0.181	.189
Event—secondary school		**6.098**	**1.405**	**<.001**	0.243	0.300	.418
Class 5 increasing absences/declining Cs							
	67/9.6%						
Intercept		**4.067**	**0.437**	**<.001**	**6.248**	**0.191**	**<.001**
Slope 1		0.531	0.460	.249	0.305	0.203	.134
Quadratic 1		0.091	0.172	.598	**−0.204**	**0.071**	**.004**
Slope 2		−0.020	2.209	.993	**−2.257**	**0.351**	**<.001**
Quadratic 2		**2.690**	**0.847**	**.002**	**0.677**	**0.141**	**<.001**
Event—secondary school		**11.345**	**1.473**	**<.001**	**−1.650**	**0.321**	**<.001**
Class 6—chronic absences/elementary Cs/transition decline-rebound							
	23/3.3%						
Intercept		**7.459**	**1.004**	**<.001**	**6.340**	**0.336**	**<.001**
Slope 1		0.992	1.542	.520	0.394	0.361	.275
Quadratic 1		1.033	0.629	.101	**−0.273**	**0.126**	**.030**
Slope 2		−6.013	5.359	.262	0.533	1.030	.605
Quadratic 2		−0.023	1.997	.991	0.258	0.351	.463
Event—secondary school		**35.247**	**4.624**	**<.001**	**−4.464**	**0.511**	**<.001**

GPA, grade point average. Statistically significant parameter estimates are bolded. Linear slope and quadratic terms should be interpreted jointly when evaluating trajectory shape.

Three classes demonstrated stable absences in elementary school with slightly increasing absences in secondary school that were paired with relatively stable trajectories of GPA, while another three classes had chronically increasing absences over elementary and secondary school, which were paired with variable trajectories of GPA. One third of students followed a high-achieving increasing trajectory for GPA (“As”) across Grades 5–12 (*n* = 225; 32.2%) with low absences in elementary school that slightly increased across secondary school. Over one quarter of students showed a similar pattern (slight increase and relatively stable secondary school) with slightly lower intercept (“Bs”) (*n* = 203; 29.1%). The absences trajectory for this class was similar to the “As” class. Class 3 (*n* = 123; 17.6%; “B/Cs”) displayed stable elementary grades, starting at a 6.81 GPA (C+/B−) intercept, that remained stable over secondary school. Students in this class also followed a low trajectory of absences that increased over secondary school, and exceeded the chronically absent cutoff (i.e., 10%) by Grade 12.

The remaining three classes demonstrated more variable trajectories. Class 4 showed an increasing elementary absences trend with steeply increasing absences across secondary school, reaching an estimated 27% days absent by Grade 12. This pattern of absences was coupled with stable B-level GPA through elementary school and into Grade 9 which declined across the remainder of secondary school (*n* = 57; 8.2%; “increasing absences/elementary Bs, secondary school decline”). Class 5 showed low-stable elementary school absences, increasing significantly over the transition, with quadratic growth in secondary school, reaching 40% days absent by Grade 12. This was paired with declining C-level grades in elementary school that continued through Grade 11, increasing slightly in Grade 12 (*n* = 67; 9.6%; “increasing absences/declining Cs”). Class 6 displayed chronically increasing absences across elementary school, with a marked increase in Grade 8 to Grade 9, before declining across secondary school. The trend for elementary school GPA was like Class 5; however, they exhibited more than a full letter grade drop in GPA from Grade 8–9 before increasing to nearly Grade 5 levels in Grade 12 (*n* = 23; 3.3%; “chronically absent/elementary Cs/transition decline-rebound”).

#### 3.4.1 Class validation

To substantiate the class structure, we examined differences between academic functioning classes on parent-reported absences in Grades 6–12 and standardized test scores in Grade 6 and Grade 9. The classes differed on parent-reported days absent in all seven years examined, *p*s < .001. In Grade 6 and Grade 7, the three stable absences classes and the increasing absences/declining Cs classes did not differ and were lower than the remaining two classes with elevated absences and changed over time in line with the estimated proportion of days. In Grade 9 and 10, the increasing absences/elementary Bs- secondary school decline and increasing absences/declining Cs classes were higher than the stable absences groups, and lower than the elevated trajectory class. In Grades 11 and 12, there were no differences between the three elevated trajectories on parent-reported absences. The classes also differed on EQAO scores in Grade 6 reading, *F*(5, 666) = 84.585, *p* < .001, writing, *F*(5, 668) = 67.946, *p* < .001, and math, *F*(5,664) = 72.001, *p* < .001. For all subjects, the Stable absences/As class reported the highest scores, followed by the Stable absences/Bs class, then the Increasing absences/Bs- secondary school decline class (writing scores not different). All classes with increasing/chronic absences scored similarly on math and writing, with the Stable absences/B/Cs class outperforming the chronic absences/elementary Cs-transition decline-rebound class. Classes also differed on the Grade 9 standardized math assessment, *F*(5,668) = 45.316, *p* < .001, with Stable absences/As and Stable absences/Bs achieving higher scores than the other four classes.

#### 3.4.2 Class correlates

All results are presented in Table 5. The Benjamini-Hochberg correlation was performed on Omnibus chi-square and F statistics (no changes) and for pairwise comparisons, where 14 of the 38 statistically significant (i.e., *p* < .05) comparisons were corrected and no longer considered statistically significant.

**Table 5:** Class associations.

					Percentage/Mean					
Correlates	*χ* ^2^ */F*	*df*	*p*	Phi/Eta-squared	C1	C2	C3	C4	C5	C6
					Stable absences	Stable absences	Stable absences	Increasing absences	Increasing absences	Chronic absences
					As (32.2%)	Bs (29.1%)	B/Cs (17.6%)	Elementary Bs secondary school decline (8.2%)	Declining Cs (9.6%)	Elementary Cs transition decline-rebound (3.3%)
Sex (% girls)^1^	48.221	5	<.001	.263	68.4%^a^	49.3%^b^	31.7%^c^	57.9%^ab^	43.3%^bc^	56.5%^ab^
Ethnicity (% White)^1^	4.850	5	.435	.087	85.2%	82.5%	76.1%	84.6%	79.3%	85.0%
Middle school^a^	6.036	5	.303	.093	48.7%	51.0%	39.3%	52.6%	41.8%	52.4%
Household income (Grade 5)	18.237	5	<.001	.124	6.91^a^	6.51^a^	5.48^b^	5.66^ab^	5.13^c^	3.43ᶜ
Parental education (Grade 5)^2^	28.047	5	<.001	.174	3.60^a^	3.29^b^	2.84^c^	2.64^cd^	2.52^cd^	2.18^d^
Bullying victimization (Grades 5–8)	8.216	5	<.001	.056	0.54^a^	0.69^ab^	0.73^ab^	0.67^ab^	0.95^b^	1.09^ab^
Bullying victimization (Grades 9–12)	3.589	5	.003	.033	0.32^a^	0.37^a^	0.30^a^	0.41^a^	0.55^a^	0.52^a^
Depression symptoms (Grades 5–8)	10.258	5	<.001	.069	3.26^a^	4.37^ab^	4.98^bc^	4.57^abc^	6.26^bc^	8.52ᶜ
Depression symptoms (Grades 9–12)	4.945	5	<.001	.044	4.30^a^	4.68^a^	5.38^a^	7.37^a^	5.96^a^	9.33^a^
Anxiety symptoms (Grades 5–8)	2.083	5	.066	.015	7.09	7.51	7.93	8.29	8.72	8.88
Anxiety symptoms (Grades 9–12)^2^	3.146	5	.008	.029	9.93^ab^	9.56^ab^	8.68^a^	11.98^b^	9.16^abc^	14.02^bc^

Pair-wise comparisons not examined for non-significant tests. Comparisons adjusted for multiple testing. Each superscript letter within a row denotes a subset of academic functioning categories whose proportions or means levels do not differ significantly from each other, *p>*.05, and after applying the Benjamini -Hochberg correction.

^1^ Chi-square, all other results are ANOVA.

^2^ Levene's statistic, *p* > .05, LSD *post-hoc* pair-wise comparisons, all others are Dunnet's T3.

##### 3.4.2.1 Demographics

Gender was significantly associated with class membership with a large effect size (*φ* = .263), with more girls than expected (standardized residual = 3.2) and fewer boys than expected (standardized residual = −3.4) in the Stable absences/As class and fewer girls expected (standardized residual = −3.2) and more boys than expected (standardized residual = 3.4) in the Stable absences/B/Cs class. Race/ethnicity and elementary school type (Kindergarten to Grade 8 vs. middle school) were not associated with class membership. Classes also differed by household income (moderate-large effect; *η*^2^ = .124). The “Chronic absences/elementary Cs transition decline-rebound” class had the lowest household income (exception “Increasing absences/Declining Cs”). For parental education, Stable absences/As and Stable absences/Bs had higher parental education than other classes (large effect; *η*^2^ = .174).

##### 3.4.2.2 Bullying victimization

Although statistically significant omnibus tests were found for bullying victimization in elementary and secondary school, the effect sizes were small. The Levene's test of homogeneity of variance was statistically significant, and pairwise comparisons were completed with the Dunnet T3 test. Following the BH corrections for multiple testing, in elementary school, the Stable absences/As class reported lower bullying victimization compared to the Increasing absences/declining Cs class. Although the Chronic absences/elementary Cs-transition decline-rebound class reported the highest victimization rates, the relatively higher standard errors likely precluded detection of a statistically significant result. There were no differences in secondary school bullying victimization scores.

##### 3.4.2.3 Mental health symptoms

The six academic functioning classes were found to differ on mental health symptomology, with small to medium effect sizes (*η*^2^ = .015-.069). In elementary school, the Stable absences/As class reported lower depression scores compared to the Stable absences/B/Cs. The Chronic absences/elementary Cs transition decline-rebound class reported higher depression symptoms than the Stable absences/As and Stable absences/Bs classes. There were no differences in anxiety symptoms in elementary school. In secondary school, there were no statistically significant pairwise comparisons between classes for depression symptoms. The Stable absences/B/Cs class reported lower secondary school anxiety symptoms than the “Increasing absences/Elementary Bs secondary school decline” and the “Chronic absences/Elementary Cs transition decline-rebound” classes.

### 3.5 Sensitivity analysis

Classes were estimated using a subset of participants who had data in elementary school and in secondary school (*n* = 573). The structure and trajectories of academic functioning classes replicated those found with the full sample. Specifically, class sizes were within 0.17%-2.77% of classes estimated using FILM with participants with any data see Supplementary Figure S1.

## 4 Discussion

### 4.1 Summary of Key findings, interpretation, and integration

We followed a large cohort of Canadian students from Grades 5 through 12 to examine the co-development of school absences and academic achievement, with attention to changes surrounding the secondary school transition from Grade 8 to Grade 9. On average, absences increased, and GPA remained relatively stable across the eight-year period, with the steepest changes emerging after entry to secondary school. Using piecewise latent growth models, we found a significant discontinuity in absences at Grade 9; compared to Grade 8, students missed approximately 3% more instructional days in the first year of secondary school which was followed by a continued upward trend through Grade 12. GPA trajectories, by contrast, were stable across elementary school, and declined only modestly in secondary school. We also observed that higher percent days absent and increasing absences trajectories were associated with lower GPAs and declining GPA trajectories. Further, significant variation in parameter estimates indicated heterogeneity in trajectories of absences and GPAs.

Person-centered analyses revealed six distinct joint trajectories of absences and GPA. Most students were in one of three groups that exhibited consistently low elementary school absences, increasing slightly in secondary school and relatively stable academic achievement with high (As), moderate (Bs), or mid-range (B/Cs) GPAs. The remaining one-fifth of students were classified in one of three groups demonstrating chronic or increasing absences, coupled with distinct GPA trajectories: (1) increasing absences with stable elementary B-level GPAs declining in secondary school, (2) increasing absences with declining Cs, and (3) chronic absences with elementary C-level GPAs with transition decline–rebound. Class membership was differentiated by gender, socioeconomic status (SES), and mental health symptoms. Girls were overrepresented in the stable absences/stable As class, and boys were overrepresented in the stable absences/stable B/Cs class. Students in the increasing absences/declining GPA classes had the lowest SES. The chronic increasing absences class had the highest depression symptoms scores in elementary school (compared to stable absences/As and stable absences Bs) with a trend in secondary school, and higher anxiety symptoms in secondary school compared to the stable B/Cs class. These results support that absences and academic achievement are developmentally intertwined and that the transition to secondary school marks a turning point in this coupling, particularly for students already showing early risk indicators.

Our results converge with international findings showing increased school absenteeism and, for some students, academic difficulties during secondary school transitions (24, 26, 70). Both domains demonstrated the characteristic pattern of positive, stable functioning in elementary school followed by divergence and widening disparities during secondary school. Although there are no published Canadian longitudinal studies for comparison, our average trajectory for absences appears remarkably similar to the cross-sectional absences by grade figure published by the Toronto District School Board (42). These findings extend the robust pattern of transition-linked academic vulnerability identified in U.S. and European samples (30, 46, 49) to a Canadian context where such longitudinal data have been lacking.

In the absence of comparable joint-trajectory studies, our results are consistent with prior research on single construct trajectories indicating that most students maintained low absence levels (46, 48, 49), whereas a subset displayed escalating or chronic absenteeism (1, 47, 50). Our GPA trajectories align with prior research showing most students followed high- or moderate-stable pathways, whereas a smaller proportion showed consistently low or declining grades (54, 58–60). The three stable GPA groups in our study (A, B, and B/C level) correspond to these normative patterns, while the declining and chronically low trajectories follow risk groups reported in U.S. and Chinese samples (45, 56). Extending prior short-term studies, our eight-year data show that GPA recovery after decline was uncommon, indicating that achievement gaps often consolidate following the high-school transition. The chronic absences class showed notable variation in secondary school in both absences and GPA, which fluctuated in tandem. This small class, representing 3% of the sample, did demonstrate a coupled pattern of decline in academic functioning followed by improvement; however, the class had trajectory parameter estimates with high standard errors relative to other classes, leading us to use caution interpreting findings involving this class. It is possible that this class was composed of students who were absent from school for a variety of reasons (e.g., high medical needs; severe truancy) and encompassed heterogeneity within itself, which could contribute to the lack of differences in examining class correlates. For example, Dräger et al. (1) found separate classes with strongly increasing authorised absences (1.62%) and strongly increasing unauthorised absences (0.82%).

Replicating prior findings, we found gender differences by class membership, aligning with Alhadabi and Li (59), who found higher and more stable achievement trajectories among girls. Our results also support international evidence that SES remains a central determinant of academic functioning (64, 68), underscoring the need to address structural inequities that contribute to attendance-related disengagement. Unlike others who have found that racially/ethnically diverse students were more likely to follow at-risk academic trajectories of absences (46, 47, 50) or grades (59), we found that academic functioning classes did not vary by race/ethnicity. It is possible that findings may have been different if we had accounted for the school context of racial composition. Minority students do better after school transition when they are in a school with a higher proportion of same race/ethnic peers (24, 30). Findings may also have been masked due to our crude classification of White and racially/ethnically diverse backgrounds (an analytic decision based on low representation of several minorities) or by failure to consider other factors that impact Canadian children, such as rurality. In Canada, territorial findings by the Department of Education Government of Yukon (85) indicate that half (51%) of students missed 20 or more days of school, and this was especially problematic for rural elementary (74%) and rural secondary (80%) students with similar rates reported by students who identified as Yukon First Nation (79% overall; 77% elementary; 81% secondary). More research is needed on the nuances of the Canadian landscape.

Beyond demographic factors, psychosocial correlates further differentiated academic functioning classes, although their effects were comparatively modest. Consistent with research linking peer difficulties and emotional problems to school engagement (7, 72, 73, 86–88), students in classes marked by increasing or chronic absences and declining grades reported higher depression and, to a lesser extent, anxiety symptoms. Surprisingly, larger effects between groups were not found, given the consistent small to moderate effects found between emotional symptoms and academic functioning (72, 73, 89). Differences in bullying victimization were small but followed the same gradient, albeit few statistically significant comparisons, with higher victimization in lower-achieving, high-absence groups. By combining scores for depressive symptoms, anxiety symptoms, and bullying victimization across the entire schooling period, we may have unintentionally masked year-to-year changes that could emerge with a more fine-grained analysis, especially given the variation observed across the three at-risk classes. Finally, elementary school structure (K-8 vs. middle-school) was unrelated to trajectory membership, in contrast to studies showing that students attending middle school experience declines in grades and attendance compared to those in K-8 schools (25, 90, 91). However, others have shown that school configuration alone exerts minimal influence on academic outcomes once individual and family factors are considered (61).

Our study extends prior research in several important ways. First, to our knowledge, it is the first Canadian longitudinal analysis spanning eight years of schooling, including mid-elementary school through secondary school with official school-record data. Second, by jointly modeling absences and GPA, we provide evidence that these two indices of academic functioning co-evolve. Previous research has largely treated one as a predictor of the other [e.g., (1, 5, 62)], overlooking the possibility that the two domains of functioning are mutually reinforcing, as has been suggested (6, 44, 49). The present multi-trajectory approach revealed that students with persistently high or increasing absences rarely sustain high GPA levels, and that the combination of chronic absenteeism and declining grades often emerges around transition years. These findings also refine prior reports of transition-linked achievement declines. Whereas Benner and Graham (30) and Vásquez-Salgado and Chavira (32) documented sharp drops in grades at the transition, our results indicate this was true for a small group of students. The findings suggest that the move from elementary to secondary school may represent a sensitive period for some students, especially those already experiencing socioeconomic or emotional difficulties.

### 4.2 Mechanisms and explanatory frameworks

The study findings support conceptualizing academic functioning as a single system of engagement and achievement, rather than separate constructs. For some students, changes in function coincided with the secondary school transition. The observed patterns here can be interpreted using complementary frameworks. From a dynamic systems perspective (92, 93), absences and GPA form interdependent subsystems within a larger network of academic functioning. Perturbations at the school transition, such as increased academic demands, reduced teacher continuity, and changes in the peer group, can destabilize established patterns and prompt reorganization toward new equilibrium states such as resilience, disengagement, or re-engagement. As an example, in the chronic absence/transition decline–rebound class, students’ absences spiked at the transition but partially recovered by Grade 12, suggesting potential re-stabilization following adaptation. Other students were more resilient to the school transition, following developmentally appropriate pathways of attendance and stable achievement. Alternatively, the secondary school transition can be viewed as a transition-linked turning point, that is, the overlap of a significant life event (i.e., the transition into secondary school) on a developmental process (i.e., adolescence). This period can be a particularly vulnerable time as it also coincides with profound developmental changes in cognition, and social, emotional, and biological development (94), leading to substantial changes in psychosocial functioning in the immediate and long-term (95). Students with differing prior experiences may respond to such transitional events in varying ways including the emergence of new behaviour, discontinuation of existing behaviour, or changes in behavioural patterns (95). In the present study, most students continued on a path of developmentally normative growth in absences and maintained consistent GPAs; however, others were vulnerable to the transition, experiencing changes in their attendance and achievement.

Increased absences can disrupt instructional continuity, which in turn reduces achievement and reinforces school avoidance. This feedback loop is well documented in previous research (5, 96). Socioeconomic disparities may exacerbate this cycle by limiting access to transportation, health care, and parental monitoring (3). Mental health difficulties, particularly depressive symptoms, can further impair concentration and motivation (97), leading to both increasing absences and decreasing grades (73). Conversely, strong parental and teacher support, school belonging, and consistent expectations may sustain engagement through school transition related stress (70, 98).

### 4.3 Strengths and limitations

This study has several notable strengths. We used objective school-record data on absences and grades, minimizing recall and social desirability biases inherent in self- or parent-report measures. The eight-year longitudinal design with 4 years on either side of the secondary school transition allowed us to account for adequate time before the transition as well as how adaptation to the secondary school transition unfolded across secondary school, two notable limitations in extant school transition research (26, 70). Testing a baseline variable-centered model allowed us to examine the strength of association between curves on absences and grades. Employing parallel-process piecewise growth models and multi-trajectory analyses provided a rigorous test of whether the secondary school transition constitutes a developmental discontinuity and identified subgroups following distinct joint pathways. Class validation using parent-reported absences and standardized test scores adds convergent validity to the six-class solution. The subsample sensitivity analysis yielded comparable classes, supporting the robustness of our findings. Finally, we provide much-needed data on Canadian students (35).

Notwithstanding the notable strengths of the study, several limitations warrant consideration. First, absence records did not distinguish excused (e.g., health) vs. unexcused (e.g., skipping class) absences. Prior work shows these forms of absenteeism have differential rates of change over compulsory schooling (1) and relate differently to grades, with a higher proportion of unexcused absences more strongly related to lower test scores (96). Second, for consistency all grade data were converted to letter grades; the conversion likely reduced the variability that would have been observed in continuous percentage scores. Third, data were drawn from a single Ontario public school board, limiting generalizability to other school districts, provinces, and territories. Fourth, attrition was modest but systematic; students with lower GPAs and higher absences at baseline were underrepresented in later waves, potentially underestimating the prevalence of chronic absence trajectories. Fifth, the chronic absence class had a small sample size, and the comparison of correlates may have been underpowered to detect differences between classes. Sixth, correlates of bullying victimization and mental health symptoms were examined concurrently rather than dynamically, precluding conclusions about directionality.

### 4.4 Future directions

Future research should expand on the present findings in several ways. Although we have begun to understand how the elementary to secondary school transition influences trajectories of academic functioning, bullying victimization (69), and internalizing problems (71), it remains unclear how these areas of functioning are associated in different academic periods, particularly across school transitions. Models that parse within-person and between-person variation, such as the random-intercept cross-lagged panel model (99, 100) or the autoregressive latent trajectory model with structured residuals model (101), could test temporal precedence and cascading effects among absences, achievement, peer relationships, and emotional well-being (102). Such models allow testing of how factors may initiate a cascade of school withdrawal to declining achievement, as well as plausible mechanisms. For example, bullying victimization may prompt school refusal, leading to loneliness and internalizing symptoms, resulting in achievement declines. In addition, multi-trajectory modeling approaches with simultaneous trajectories of academic functioning, bullying victimization, and internalizing symptoms would clarify the degree to which these domains co-develop and identify distinct subgroups characterized by similar patterns of risk and adaptation across the school transition.

Future research should also examine early adult functional outcomes (e.g., post-secondary enrollment and completion, earnings, positive relationships) of differing joint trajectory classes. For example, students with declining GPAs and those with low-increasing GPAs over the first 1.5 years of secondary school were far more likely to drop out of secondary school compared to mid-achieving students (58), underscoring the long-term risks associated with these lower-achieving trajectories. Extending this work beyond a deficit-focused framework to include indicators of positive behavioural adjustment, such as prosocial behaviour and school engagement, would further help identify resilient developmental profiles, which could inform prevention and intervention programs. Incorporating reason-coded absence data (i.e., excused vs. unexcused) would allow finer-grained analyses of risk mechanisms as unexcused absences are more related to academic risk than excused (21).

As this is the first eight-year study of Canadian students’ absence and grade data, results require replication, not only with the time frame, but also in other provinces across the country. Given Canada's increasingly diverse population (103), future studies should examine cultural and regional differences, including Indigenous, First-Nations, Métis, and rural communities in northern Canada where absenteeism rates remain disproportionately high compared to non-First Nations-identifying and urban students (85). Indeed, researchers have highlighted the urgent need to understand school attendance supports among Indigenous students in Canada (104). Understanding these Canada-specific contextual factors is essential for designing equitable policy. Finally, for studies including data collected during the COVID-19 pandemic, careful attention should be given to school closures and other mitigating factors, as school absenteeism and grades were adversely affected (105–107).

### 4.5 Implications

The findings of the present study have several practical implications. At the practice level, the results underscore the importance of monitoring both school attendance and GPA as early indicators of disengagement. Schools could integrate routinely collected attendance and grade data into early warning systems to flag students exhibiting upward absence trends or GPA declines, especially during school transition years. Kearney et al. (108) highlighted the potential early warning sign that school attendance problems play in identifying students at risk for academic failure, mental health difficulties, and school dropout, emphasizing the importance of early detection and intervention to prevent these cascading adverse outcomes. At the policy level, the results reinforce calls for standardized national reporting of attendance metrics (35) to enable longitudinal tracking and cross-provincial/territorial comparisons. Finally, our findings highlight the need for multi-tiered intervention approaches (109). Universal strategies such as consistent attendance messaging and supportive school climates should be complemented by targeted supports for youth exhibiting early risk (e.g., frequent absences in late elementary school; increasing absences in early secondary school) and intensive interventions for those with chronic absence (11). Addressing SES-related disparities through equitable access to health, transportation, and academic resources remains critical (110). Several Canadian provincial governments have proposed such principles of early warning and tiered intervention through formal policy directives [e.g. (111, 112)].

## 5 Conclusion

This study provides the first Canadian longitudinal evidence that school attendance and academic achievement are developmentally intertwined across the transition to secondary school. Although most students maintain consistent attendance and achievement, a significant minority follow risk trajectories marked by increasing absences and declining GPA, particularly among students with lower SES. Students in the most at-risk classes showed slightly increased bullying victimization and internalizing symptoms. Monitoring the joint progression of school absences and achievement can help educators identify students at risk of disengagement before such patterns become entrenched. Supporting continuity of learning and connection during school transitions is therefore essential to promoting long-term educational success.

## Data Availability

The data that support the findings of this study are available on reasonable request to the corresponding author. The data are not publicly available due to privacy and ethical restrictions associated with longitudinal studies. Requests to access the datasets should be directed to tracy.vaillancourt@uottawa.ca.

## Footnotes

^1^Benner and Wang [13] modeled attendance rates (days present), not absences. For consistency, their classes are described here in terms of the absence equivalents (e.g., decreasing attendance=increasing absences).

^2^During Time 4 (Grade 8) of data collection the Ministry of Education of Ontario changed provincial report card requirements. Instead of three term reports students received a preliminary report and two official term assessments. The school board provided the research team with the two official reports in Time 4 data collection.

## References

[1] Dräger J Klein M Sosu E. Trajectories of school absences across compulsory schooling and their impact on children’s academic achievement: an analysis based on linked longitudinal survey and school administrative data. PLoS One. (2024) 19(8):e0306716. 10.1371/journal.pone.0306716 39133716 PMC11318909

[2] Keppens G. School absenteeism and academic achievement: does the timing of the absence matter? Learn Instr. (2023) 86(August):101769. 10.1016/j.learninstruc.2023.101769

[3] Klein M Sosu E. School absences, academic achievement, and adolescents’ post-school destinations. Oxford Rev Educ. (2025) 51(3):339–56. 10.1080/03054985.2024.2308520

[4] Klein M Sosu EM Dare S. School absenteeism and academic achievement: does the reason for absence matter? AERA Open. (2022) 8:23328584211071115. 10.1177/23328584211071115

[5] Liu J Lee M Gershenson S. The short- and long-run impacts of secondary school absences. J Public Econ. (2021) 199:104441. 10.1016/j.jpubeco.2021.104441

[6] Roderick M Camburn EM. Risk and recovery from course failure in the early years of high school. Am Educ Res J. (1999) 36(2):303–43. 10.3102/00028312036002303

[7] Gubbels J Van Der Put CE Assink M. Risk factors for school absenteeism and dropout: a meta-analytic review. J Youth Adolesc. (2019) 48(9):1637–67. 10.1007/s10964-019-01072-5 31312979 PMC6732159

[8] Dräger J Klein M Sosu E. The long-term consequences of early school absences for educational attainment and labour market outcomes. Br Educ Res J. (2024) 50(4):1636–54. 10.1002/berj.3992

[9] French MT Homer JF Popovici I Robins PK. What you do in high school matters: high school GPA, educational attainment, and labor market earnings as a young adult. East Econ J. (2015) 41(3):370–86. 10.1057/eej.2014.22

[10] Kearney CA. School absenteeism and school refusal behavior in youth: a contemporary review. Clin Psychol Rev. (2008) 28(3):451–71. 10.1016/j.cpr.2007.07.012 17720288

[11] Kearney CA Gonzálvez C Graczyk PA Fornander MJ. Reconciling contemporary approaches to school attendance and school absenteeism: toward promotion and nimble response, global policy review and implementation, and future adaptability (part 1). Front Psychol. (2019) 10:2222. 10.3389/fpsyg.2019.02222 31681069 PMC6805702

[12] Maravilla JC Betts KS Couto e Cruz C Alati R. Factors influencing repeated teenage pregnancy: a review and meta-analysis. Am J Obstet Gynecol. (2017) 217(5):527–545.e31. 10.1016/j.ajog.2017.04.021 28433733

[13] Sörberg Wallin A Koupil I Gustafsson J-E Zammit S Allebeck P Falkstedt D. Academic performance, externalizing disorders and depression: 26,000 adolescents followed into adulthood. Soc Psychiatry Psychiatr Epidemiol. (2019) 54(8):977–86. 10.1007/s00127-019-01668-z 30783692

[14] Bradley BJ Greene AC. Do health and education agencies in the United States share responsibility for academic achievement and health? A review of 25 years of evidence about the relationship of adolescents’ academic achievement and health behaviors. J Adolesc Health. (2013) 52(5):523–32. 10.1016/j.jadohealth.2013.01.008 23535065

[15] Gakh M Coughenour C Assoumou BO Vanderstelt M. The relationship between school absenteeism and substance use: an integrative literature review. Subst Use Misuse. (2020) 55(3):491–502. 10.1080/10826084.2019.1686021 31805820

[16] Kendler KS Ohlsson H Fagan AA Lichtenstein P Sundquist J Sundquist K. Nature of the causal relationship between academic achievement and the risk for alcohol use disorder. J Stud Alcohol Drugs. (2020) 81(4):446–53. 10.15288/jsad.2020.81.446 32800080 PMC7437558

[17] Barnert ES Perry R Shetgiri R Steers N Dudovitz R Heard-Garris NJ Adolescent protective and risk factors for incarceration through early adulthood. J Child Fam Stud. (2021) 30(6):1428–40. 10.1007/s10826-021-01954-y

[18] Epstein S Roberts E Sedgwick R Polling C Finning K Ford T School absenteeism as a risk factor for self-harm and suicidal ideation in children and adolescents: a systematic review and meta-analysis. Eur Child Adolesc Psychiatry. (2020) 29(9):1175–94. 10.1007/s00787-019-01327-3 30989389 PMC7116080

[19] Gunnell D Löfving S Gustafsson J-E Allebeck P. School performance and risk of suicide in early adulthood: follow-up of two national cohorts of Swedish schoolchildren. J Affect Disord. (2011) 131(1):104–12. 10.1016/j.jad.2011.01.002 21296426

[20] Kosik R Fan A Mandell G Su T-P Nguyen T Chen J Academic performance in childhood and the risk of attempting suicide as an adult. Eur J Psychiatry. (2017) 31(2):73–9. 10.1016/j.ejpsy.2017.03.002

[21] Henderson CM Fantuzzo JW. Challenging the core assumption of chronic absenteeism: are excused and unexcused absences equally useful in determining academic risk Status? J Educ Students Placed Risk (JESPAR). (2023) 28(3):259–93. 10.1080/10824669.2022.2065636

[22] Attendance Works. Chronic Absence (2025). Available online at: https://www.attendanceworks.org/chronic-absence/the-problem/ (Accessed October 10, 2025).

[23] Organisation for Economic Co-operation and Development (OECD). Education GPS (2023). Available online at: https://gpseducation.oecd.org/CountryProfile?primaryCountry=CAN (Accessed October 10, 2025).

[24] Benner AD. The transition to high school: current knowledge, future directions. Educ Psychol Rev. (2011) 23(3):299–328. 10.1007/s10648-011-9152-0 21966178 PMC3182155

[25] Eccles JS Lord S Midgley C. What are we doing to early adolescents? The impact of educational contexts on early adolescents. Am J Educ. (1991) 99(4):521–42. 10.1086/443996

[26] Evans D Borriello GA Field AP. A review of the academic and psychological impact of the transition to secondary education. Front Psychol. (2018) 9:1482. 10.3389/fpsyg.2018.01482 30210385 PMC6123573

[27] Spernes K. The transition between primary and secondary school: a thematic review emphasising social and emotional issues. Res Papers Educ. (2022) 37(3):303–20. 10.1080/02671522.2020.1849366

[28] Beatson R Quach J Canterford L Farrow P Bagnall C Hockey P Improving primary to secondary school transitions: a systematic review of school-based interventions to prepare and support student social-emotional and educational outcomes. Educ Res Rev. (2023) 40:100553. 10.1016/j.edurev.2023.100553

[29] Rice F Ng-Knight T Riglin L Powell V Moore G McManus IC Pupil mental health, concerns and expectations about secondary school as predictors of adjustment across the transition to secondary school: a longitudinal multi-informant study. *School Mental Health*. October. (2021) 21:279–98. 10.1007/S12310-021-09415-Z

[30] Benner AD Graham S. The transition to high school as a developmental process among multiethnic urban youth. Child Dev. (2009) 80(2):356–76. 10.1111/j.1467-8624.2009.01265.x 19466997

[31] Reyes O Gillock KL Kobus K Sanchez B. A longitudinal examination of the transition into senior high school for adolescents from urban, low-income Status, and predominantly minority backgrounds. Am J Community Psychol. (2000) 28(4):519–44. 10.1023/A:1005140631988 10965389

[32] Vasquez-Salgado Y Chavira G. The transition from middle school to high school as a developmental process among latino youth. Hisp J Behav Sci. (2014) 36(1):79–94. 10.1177/0739986313513718 PMC4155758 25202166

[33] U.S. Department of Education. Chronic Absenteeism (2025). Available online at: http://www.ed.gov/teaching-and-administration/supporting-students/chronic-absenteeism (Accessed October 10, 2025).

[34] Department for Education, Government of the United Kingdom. Working Together to Improve School Attendance (2024). Available online at: https://www.gov.uk/government/publications/working-together-to-improve-school-attendance, GOV.UK (Accessed October 6, 2025).

[35] Whitley J McBrearty N Rogers MA David Smith J. The current state of school attendance research and data in Canada. Educ Sci. (2025) 15(8):964. 10.3390/educsci15080964

[36] Organisation for Economic Co-operation and Development (OECD). PISA: Programme for International Student Assessment (2025). Available online at: https://www.oecd.org/en/about/programmes/pisa.html (Accessed October 10, 2025)

[37] Statistics Canada. National Longitudinal Survey of Children and Youth (NLSCY) (2009). Available online at: https://www23.statcan.gc.ca/imdb/p2SV.pl?Function=getSurvey&SDDS=4450 (Accessed October 9, 2025).

[38] Statistics Canada. Canadian Health Survey on Children and Youth—2023 (2023). Available online at: https://www23.statcan.gc.ca/imdb/p3Instr.pl?Function=assembleInstr&a=1&&lang=en&Item_Id=1509628#qb1514463 (Accessed October 9, 2025).

[39] Statistics Canada. Canadian Health Survey on Children and Youth—2025 (2025). Available online at: https://www23.statcan.gc.ca/imdb/p3Instr.pl?Function=assembleInstr&lang=en&Item_Id=1574659 (Accessed October 9, 2025).

[40] Birioukov A. Absent on absenteeism: academic silence on student absenteeism in Canadian education. Canad J Educ. (2021) 44(3):718–31. 10.53967/cje-rce.v44i3.4663

[41] Hancock KJ Shepherd CCJ Lawrence D Zubrick SR. Student Attendance and Educational Outcomes: every Day Counts. Canberra: Department Of Education, Employment Workplace Relations (2013). 263 pages. 10.13140/2.1.4956.6728

[42] Brown RS. Absenteeism in the TDSB. Res Today. (2009) 4(1). (2 page document). Toronto District School Board. Available online at: https://www.tdsb.on.ca/Portals/research/docs/reports/RT_Absenteeism.pdf

[43] Balfanz R Byrnes V. The Importance of Being There: A Report on Absenteeism in the Nation’s Public Schools. Baltimore: Johns Hopkins University Center for Social Organization of Schools (2012).

[44] Kieffer MJ Marinell WH Neugebauer SR. Navigating into, through, and beyond the middle grades: the role of middle grades attendance in staying on track for high school graduation. J Sch Psychol. (2014) 52(6):549–65. 10.1016/j.jsp.2014.09.002 25432271

[45] Liu Y Lu Z. Trajectories of Chinese students’ sense of school belonging and academic achievement over the high school transition period. Learn Individ Differ. (2011) 21(2):187–90. 10.1016/j.lindif.2010.12.007

[46] Wei W. Exploring patterns of absenteeism from prekindergarten through early elementary school and their associations with children’s academic outcomes. AERA Open. (2024) 10:23328584241228212. 10.1177/23328584241228212

[47] Simon O Nylund-Gibson K Gottfried M Mireles-Rios R. Elementary absenteeism over time: a latent class growth analysis predicting fifth and eighth grade outcomes. Learn Individ Differ. (2020) 78:101822. 10.1016/j.lindif.2020.101822

[48] Tunkkari M Kiuru N Virtanen T Vasalampi K. Antecedents of developmental trajectories of school absences among adolescents. Scand J Educ Res. (2025):1–14. 10.1080/00313831.2025.2506380

[49] Schoeneberger JA. Longitudinal attendance patterns: developing high school dropouts. Clear House. (2012) 85(1):7–14. 10.1080/00098655.2011.603766

[50] Benner AD Wang Y. Shifting attendance trajectories from middle to high school: influences of school transitions and changing school contexts. Dev Psychol. (2014) 50(4):1288–301. 10.1037/a0035366 24364827 PMC3981879

[51] Espy KA Fang H Charak D Minich N Gerry Taylor H. Growth mixture modeling of academic achievement in children of varying birth weight risk. Neuropsychology. (2009) 23(4):460–74. 10.1037/a0015676 19586210 PMC2776698

[52] Fu R Chen X Wang L Yang F. Developmental trajectories of academic achievement in Chinese children: contributions of early social-behavioral functioning. J Educ Psychol. (2016) 108(7):1001–12. 10.1037/edu0000100

[53] Lee J. Tripartite growth trajectories of Reading and math achievement: tracking national academic progress at primary, middle, and high school levels. Am Educ Res J. (2010) 47(4):800–32. 10.3102/0002831210365009

[54] Lu Y. Modeling math growth trajectory—an application of conventional growth curve model and growth mixture model to ECLS K-5 data. J Educ Iss. (2016) 2(1):166. 10.5296/jei.v2i1.9197

[55] Akos P Rose RA Orthner D. Sociodemographic moderators of middle school transition effects on academic achievement. J Early Adolesc. (2015) 35(2):170–98. 10.1177/0272431614529367

[56] Nie Q Teng Z Yang C Lu X Liu C Zhang D Psychological suzhi and academic achievement in Chinese adolescents: a 2-year longitudinal study. Br J Educ Psychol. (2021) 91(2):638–57. 10.1111/bjep.12384 33118619

[57] Duchesne S Larose S Guay F Vitaro F Tremblay R. The transition from elementary to high school: the pivotal role of mother and child characteristics in explaining trajectories of academic functioning. Int J Behav Dev. (2005) 29(5):409–17. 10.1080/01650250500206067

[58] Bowers AJ Sprott R. Examining the multiple trajectories associated with dropping out of high school: a growth mixture model analysis. J Educ Res. (2012) 105(3):176–95. 10.1080/00220671.2011.552075

[59] Alhadabi A Li J. Trajectories of academic achievement in high schools: growth mixture model. J Educ Iss. (2020) 6(1):140–65. 10.5296/jei.v6i1.16775

[60] Fu YC Chen SL Quetzal AS Lee HM Lin YH. Group-Based trajectory model to analyze the growth of Students’ academic performance: a longitudinal investigation at one Taiwanese high school. Asia Pac Educ Rev. (2022) 23(3):515–26. 10.1007/s12564-022-09792-3

[61] Schwerdt G West MR. The impact of alternative grade configurations on student outcomes through middle and high school. J Public Econ. (2013) 97:308–26. 10.1016/j.jpubeco.2012.10.002

[62] Dräger J Klein M Sosu E. Does the impact of pupil absences on achievement depend on their timing? Am Educ Res J. (2025) 62(5):872–908. 10.3102/00028312251347666

[63] Cattan S Kamhöfer DA Karlsson M Nilsson T. The long-term effects of student absence: evidence from Sweden. The Economic Journal. (2023) 133(650):888–903. 10.1093/ej/ueac078

[64] Klein M Sosu EM. School attendance and academic achievement: understanding variation across family socioeconomic Status. Sociol Educ. (2024) 97(1):58–75. 10.1177/00380407231191541

[65] Akos P Galassi JP. Middle and high school transitions as viewed by students, parents, and teachers. Prof School Counseling. (2004) 7(4):212–21.

[66] Wade M Prime H Browne DT. Why we need longitudinal mental health research with children and youth during (and after) the COVID-19 pandemic. Psychiatry Res. (2020) 290:113143. 10.1016/j.psychres.2020.113143 32502829 PMC7253952

[67] Symonds JE Galton M. Moving to the next school at age 10–14 years: an international review of psychological development at school transition. Review of Education. (2014) 2(1):1–27. 10.1002/rev3.3021

[68] Caro DH McDonald JT Douglas Willms J. Socio-economic status and academic achievement trajectories from childhood to adolescence. Canad J Educ. (2009) 32(3):558–90.

[69] Vaillancourt T Brittain H Farrell AH Krygsman A Vitoroulis I. Bullying involvement and the transition to high school: a brief report. Aggress Behav. (2023) 49(4):409–17. 10.1002/ab.22082 36916023

[70] Jindal-Snape D Hannah EFS Cantali D Barlow W MacGillivray S. Systematic literature review of primary‒secondary transitions: international research. Review of Education. (2020) 8(2):526–66. 10.1002/rev3.3197

[71] Brittain H Vaillancourt T. Childhood to Adolescence Co-Occurring Depression and Anxiety Trajectories Across School Transition: Early Adulthood Mental Health and Functional Outcomes (2025). Under review.

[72] Finning K Ukoumunne OC Ford T Danielsson-Waters E Shaw L Romero De Jager I Review: the association between anxiety and poor attendance at school—a systematic review. Child Adolesc Ment Health. (2019) 24(3):205–16. 10.1111/camh.12322 32677217

[73] Finning K Ukoumunne OC Ford T Danielsson-Waters E Shaw L Romero De Jager I The association between child and adolescent depression and poor attendance at school: a systematic review and meta-analysis. J Affect Disord. (2019) 245:928–38. 10.1016/j.jad.2018.11.055 30699878

[74] Högberg B Strandh M Petersen S Nilsson K. Associations between academic achievement and internalizing disorders in Swedish students aged 16 years between 1990 and 2018. Eur Child Adolesc Psychiatry. (2025) 34(5):1661–71. 10.1007/s00787-024-02597-2 39470790 PMC12122550

[75] Rogers MA Klan A Oram R Krause A Whitley J Smith DJ School absenteeism and child mental health: a mixed-methods study of internalizing and externalizing symptoms. School Ment Health. (2024) 16(2):331–42. 10.1007/s12310-024-09640-2

[76] Queen’s Printer for Ontario. EQAO: Ontario’s Provincial Assessment Program: Its History and Influence, 1996-2012. Toronto: Education Quality and Accountability Office (2013).

[77] Reynolds CR Kamphaus RW. Behavior Assessment System for Children. 2nd Ed Pearson. Bloomington, MN: Pearson Assessments (2004).

[78] Krygsman A Vaillancourt T. Longitudinal associations between depression symptoms and peer experiences: evidence of symptoms-driven pathways. J Appl Dev Psychol. (2017) 51:20–34. 10.1016/j.appdev.2017.05.003

[79] Olweus D. The Revised Olweus Bully/Victim Questionnaire. Bergen, Norway: University of Bergen (1996).

[80] Vaillancourt T Trinh V McDougall P Duku E Cunningham L Cunningham C Optimizing population screening of bullying in school-aged children. J Sch Violence. (2010) 9(3):233–50. 10.1080/15388220.2010.483182

[81] West SG McNeish D Savord A. Model fit in structural equation modeling. In: Hoyle RH, editor. Handbook of Structural Equation Modeling. Guilford Press (2023) 2nd Ed. p. 184–205.

[82] Nagin DS Jones BL Passos VL Tremblay RE. Group-Based multi-trajectory modeling. Stat Methods Med Res. (2018) 27(7):2015–23. 10.1177/0962280216673085 29846144

[83] Benjamini Y Hochberg Y. “Controlling the false discovery rate: a practical and powerful approach to multiple testing.”. J R Stat Soc B. (1995) 57(1):289–300. 10.1111/j.2517-6161.1995.tb02031.x

[84] Enders CK. Applied Missing Data Analysis. 2nd Ed New York: The Guilford Press (2022).

[85] Department of Education, Government of Yukon. Yukon Wide Student Data Report—School Year 2023–24 (2025). Available online at: https://yukon.ca/sites/default/files/2025-01/edu-Yukon-wide-report-2023-24.pdf (Accessed October 6, 2025).

[86] Moore SE Norman RE Suetani S Thomas HJ Sly PD Scott JG. Consequences of bullying victimization in childhood and adolescence: a systematic review and meta-analysis. World J Psychiatry. (2017) 7(1):60–76. 10.5498/wjp.v7.i1.60 28401049 PMC5371173

[87] Nakamoto J Schwartz D. Is peer victimization associated with academic achievement? A meta-analytic review. Soc Dev. (2010) 19(2):221–42. 10.1111/j.1467-9507.2009.00539.x

[88] Samara M Da Silva Nascimento B El-Asam A Hammuda S Khattab N. How can bullying victimisation lead to lower academic achievement? A systematic review and meta-analysis of the mediating role of cognitive-motivational factors. Int J Environ Res Public Health. (2021) 18(5):2209. 10.3390/ijerph18052209 33668095 PMC7967665

[89] Bücker S Nuraydin S Simonsmeier BA Schneider M Luhmann M. Subjective well-being and academic achievement: a meta-analysis. J Res Pers. (2018) 74:83–94. 10.1016/j.jrp.2018.02.007

[90] Alspaugh JW. Achievement loss associated with the transition to middle school and high school. J Educ Res. (1998) 92(1):20–5. 10.1080/00220679809597572

[91] Weiss CC Christine Baker-Smith E. Eighth-grade school form and resilience in the transition to high school: a comparison of middle schools and K-8 schools. J Res Adolesc. (2010) 20(4):825–39. 10.1111/j.1532-7795.2010.00664.x

[92] Granic I Hollenstein T. A survey of dynamic systems methods for developmental psychopathology. In: Cicchetti D Cohen DJ, editors. Developmental Psychopathology. 1st Ed Wiley. Hoboken, NJ: Wiley (2015) p. 889–930. 10.1002/9780470939383.ch22

[93] Thelen E Smith LB. A Dynamic Systems Approach to the Development of Cognition and Action. Cambridge, MA: MIT Press (1994).

[94] Best O Ban S. Adolescence: physical changes and neurological development. Br J Nurs. (2021) 30(5):272–75. 10.12968/bjon.2021.30.5.272 33733842

[95] Graber JA Brooks-Gunn J. Transitions and turning points: navigating the passage from childhood through adolescence. Dev Psychol. (1996) 32(4):768–76. 10.1037/0012-1649.32.4.768

[96] Gottfried MA. Excused versus unexcused: how student absences in elementary school affect academic achievement. Educ Eval Policy Anal. (2009) 31(4):392–415. 10.3102/0162373709342467

[97] Grahek I Shenhav A Musslick S Krebs RM Koster EHW. Motivation and cognitive control in depression. Neurosci Biobehav Rev. (2019) 102(July):371–81. 10.1016/j.neubiorev.2019.04.011 31047891 PMC6642074

[98] Barber BK Olsen JA. Assessing the transitions to middle and high school. J Adolesc Res. (2004) 19(1):3–30. 10.1177/0743558403258113

[99] Mulder JD Hamaker EL. Three extensions of the random intercept cross-lagged panel model. Struct Equ Model. (2021) 28(4):638–48. 10.1080/10705511.2020.1784738

[100] Hamaker EL Kuiper RM Grasman RPPP. A critique of the cross-lagged panel model. Psychol Methods. (2015) 20(1):102–16. 10.1037/a0038889 25822208

[101] Curran PJ Howard AL Bainter SA Lane ST McGinley JS. The separation of between-person and within-person components of individual change over time: a latent curve model with structured residuals. J Consult Clin Psychol. (2014) 82(5):879–94. 10.1037/a0035297 24364798 PMC4067471

[102] Brittain H Vaillancourt T. Longitudinal associations between academic achievement and depressive symptoms in adolescence: methodological considerations and analytical approaches for identifying temporal priority. In: Lemonda CS Lockman JJ, editors. Advances in Child Development and Behavior, vol. 64. Cambridge, MA: Academic Press (2023) p. 327–55. 10.1016/bs.acdb.2022.11.003 37080673

[103] Statistics Canada. Canada in 2041: A Larger, More Diverse Population with Greater Differences Between Regions (2022). Available online at: https://www150.statcan.gc.ca/n1/en/daily-quotidien/220908/dq220908a-eng.pdf?st=uKbUpdB (Accessed October 6, 2025).

[104] Rogers M Aglukark K. Supporting school attendance among indigenous children and youth in Canada: a rapid review and call to action. First Peoples Child Fam Rev. (2024) 19(1):32–46. 10.7202/1114911ar

[105] Engzell P Frey A Verhagen MD. Learning loss due to school closures during the COVID-19 pandemic. Proc Natl Acad Sci USA. (2021) 118(17):e2022376118. 10.1073/pnas.2022376118 33827987 PMC8092566

[106] Kuhfeld M Soland J Tarasawa B Johnson A Ruzek E Liu J. Projecting the potential impact of COVID-19 school closures on academic achievement. Educ Res. (2020) 49(8):549–65. 10.3102/0013189X20965918

[107] Maldonado JE De Witte K. The effect of school closures on standardised student test outcomes. Br Educ Res J. (2022) 48(1):49–94. 10.1002/berj.3754

[108] Kearney CA Dupont R Fensken M Gonzálvez C. School attendance problems and absenteeism as early warning signals: review and implications for health-based protocols and school-based practices. Front Educ. (2023) 8:1253595. 10.3389/feduc.2023.1253595

[109] Graczyk PA Kearney CA. Roadmap for implementing a multi-tiered system of supports framework to improve school attendance. Curr Psychol. (2024) 43(17):15286–307. 10.1007/s12144-023-05478-0

[110] Sosu EM Dare S Goodfellow C Klein M. Socioeconomic Status and school absenteeism: a systematic review and narrative synthesis. Rev Educ. (2021) 9(3):e3291. 10.1002/rev3.3291

[111] Government of Manitoba. School Attendance | Manitoba Education and Early Childhood Learning (2025). Available online at: https://www.edu.gov.mb.ca/k12//attendance/policies.html (Accessed October 10, 2025).

[112] Nova Scotia Department of Education and Early Childhood Development. Student Attendance and Engagement Policy (2025). Available online at: https://www.ednet.ns.ca/student-attendance-and-engagement-policy (Accessed October 10, 2025).

[113] Rioux C Stickley ZL Little TD. Solutions for latent growth modeling following COVID-19-related discontinuities in change and disruptions in longitudinal data collection. Int J Behav Dev. (2021) 45(5):463–73. 10.1177/01650254211031631

