# Supplementary material for: School attendance matters: co-occurring trajectories of school attendance and academic achievement from elementary to secondary school in a Canadian context
Source: Front Child Adolesc Psychiatry. 2026 Mar 3;5:1729744. doi: 10.3389/frcha.2026.1729744 (PMC12992258; doi:10.3389/frcha.2026.1729744)
Supplement: Supplementary file 1 [file Datasheet1.docx]

Supplementary Material

# Supplementary Figures


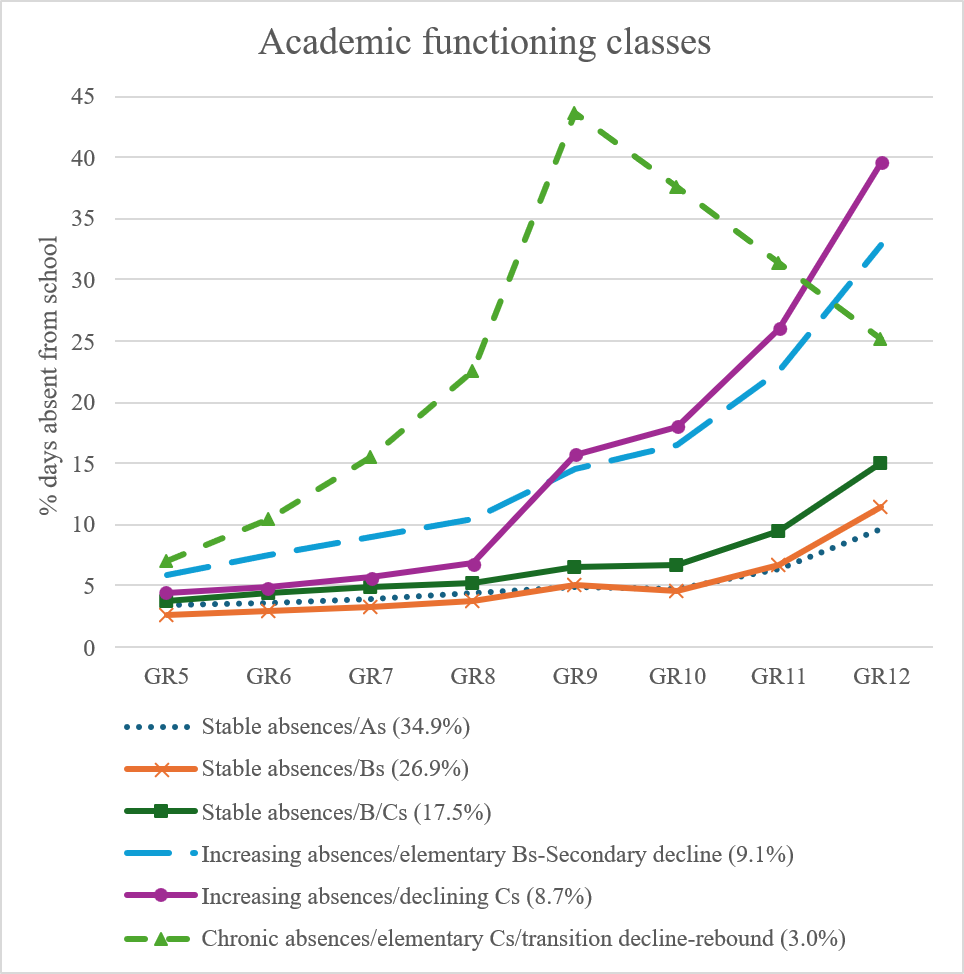

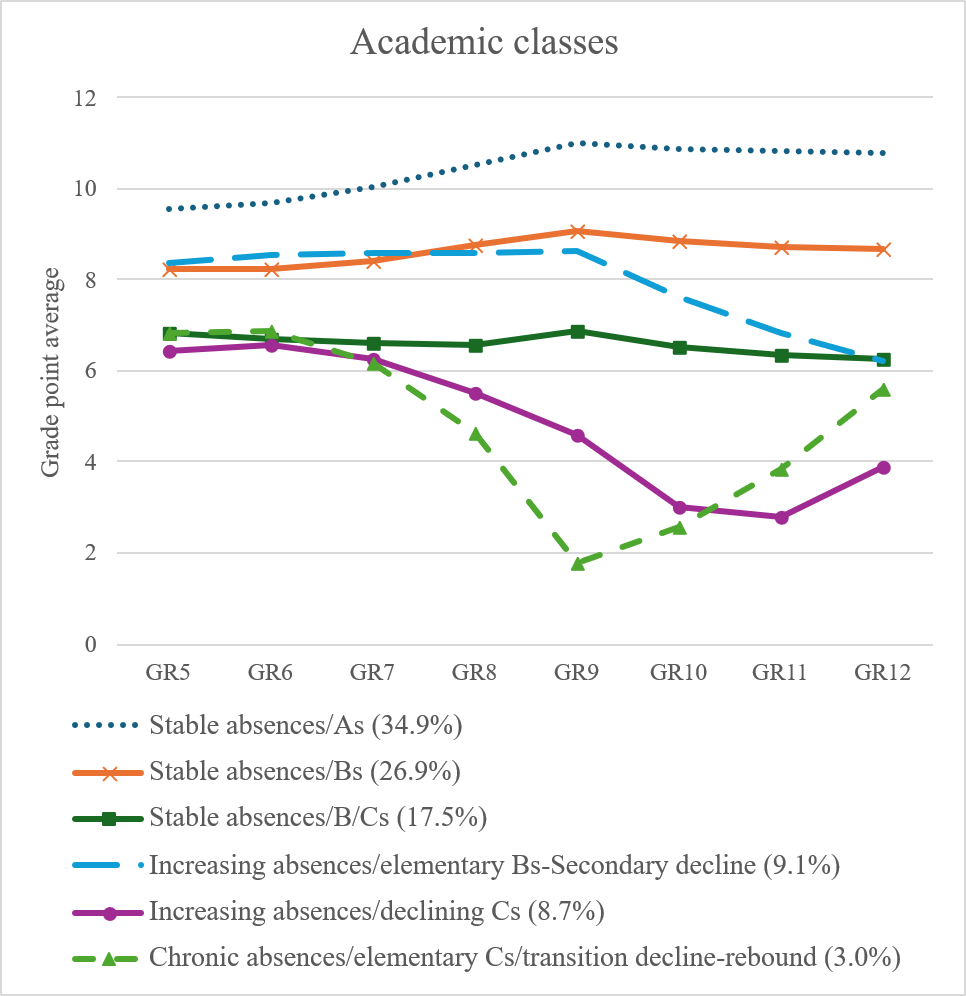


**Supplementary Figure 1. Sensitivity analysis Absences and GPA Parallel-Process Discontinuity Latent Class Growth Curve Analysis.** GR=Grade. GPA=Grade point average. Percent days absent and GPA parallel-process latent class growth analysis six class solution using a sub-sample with data in both elementary and secondary school (*n*=573). Grades 5 to 8 are elementary school and Grades 9 to 12 are secondary school.
